# Population pharmacokinetics of methotrexate and 7-hydroxymethotrexate and delayed excretion in infants and young children with brain tumor*

**DOI:** 10.1016/j.ejps.2023.106669

**Published:** 2023-12-08

**Authors:** Olivia Campagne, Jie Huang, Tong Lin, Wilburn E. Reddick, Nicholas S. Selvo, Arzu Onar-Thomas, Deborah Ward, Giles Robinson, Amar Gajjar, Clinton F. Stewart

**Affiliations:** a Department of Pharmacy and Pharmaceutical Sciences, St. Jude Children’s Research Hospital, 262 Danny Thomas Place, Memphis, TN 38105, USA; b Department of Biostatistics, St. Jude Children’s Research Hospital, Memphis, TN 38105, USA; c Department of Diagnostic Imaging, St. Jude Children’s Research Hospital, Memphis, TN 38105, USA; d Department of Oncology, St. Jude Children’s Research Hospital, Memphis, TN 38105, USA

**Keywords:** Infants, Methotrexate, 7-hydroxymethotrexate, Population pharmacokinetics, Brain tumor, Delayed excretion

## Abstract

**Purpose:**

The objectives of this study were to develop a population pharmacokinetic model of methotrexate (MTX) and its primary metabolite 7-hydroxymethotrexate (7OHMTX) in children with brain tumors, to identify the sources of pharmacokinetic variability, and to assess whether MTX and 7OHMTX systemic exposures were related to toxicity.

**Methods:**

Patients received 2.5 or 5 g/m^2^ MTX as a 24-hour infusion and serial samples were analyzed for MTX and 7OHMTX by an LC-MS/MS method. Pharmacokinetic parameters were estimated using nonlinear mixed-effects modeling. Demographics, laboratory values, and genetic polymorphisms were considered as potential covariates to explain the pharmacokinetic variability. Association between MTX and 7OHMTX systemic exposures and MTX-related toxicities were explored using random intercept logistic regression models.

**Results:**

The population pharmacokinetics of MTX and 7OHMTX were adequately characterized using two-compartment models in 142 patients (median 1.91 y; age range 0.09 to 4.94 y) in 513 courses. The MTX and 7OHMTX population clearance values were 4.6 and 3.0 l/h/m^2^, respectively. Baseline body surface area and estimated glomerular filtration rate were significant covariates on both MTX and 7OHMTX plasma disposition. Pharmacogenetic genotypes were associated with MTX pharmacokinetic parameters but had only modest influence. No significant association was observed between MTX or 7OHMTX exposure and MTX-related toxicity.

**Conclusions:**

MTX and 7OHMTX plasma disposition were characterized for the first time in young children with brain tumors. No exposure-toxicity relationship was identified in this study, presumably due to aggressive clinical management which led to a low MTX-related toxicity rate.

## 1. Introduction

In children and adolescents less than 19 years of age, brain and other tumors of the central nervous system (CNS) are the most common solid tumor and account for most of the cancer deaths in this age group (Curtin et al., 2016). Contemporary therapy for tumors of the CNS consists of maximal safe surgical resection, craniospinal irradiation (CSI), and chemotherapy; however, younger patients (e.g., < 3 years at diagnosis) often suffer debilitating adverse effects from the CSI. For example, many clinical trials for infants and young children with medulloblastoma will either omit or delay CSI, and because of this and inherent limitations in the biology of these tumors, these children often have poor prognosis. To avoid this potential poor prognosis and to limit long-term sequelae of CSI, investigators have chosen to use intensive adjuvant chemotherapy regimens instead of CSI. Recent studies have shown that the antifolate methotrexate (MTX) has been a key component of these regimens (Robinson et al., 2018).

To achieve therapeutic CNS MTX exposures, investigators have utilized high-dose methotrexate (HDMTX) with dosages > 500 mg/m^2^. A well-known risk with HDMTX therapy is the potential for delayed MTX excretion which can lead to multiple toxicities and thus requires close monitoring and leucovorin rescue. Understanding factors contributing to this phenomenon is important to both efficacy and safety in each patient. In a previous analysis, we have shown that intracranial post-resection fluid collections were associated with delayed MTX excretion in young children (Wright et al., 2015). Additionally, we have recently shown MTX clearance in infants ≤ 31 days at enrollment on study was approximately half that of older infants. This led to a dosage recommendation to decrease the dosage in that patient population by one-half (i.e., 2.5 g/m^2^) compared to older patients that would receive the full dosage or 5 g/m^2^ (Panetta et al., 2020).

Large inter-patient variability in MTX disposition has been observed in infants and young children with brain tumors and a large percentage of this is currently unexplained (Panetta et al., 2020; Shkalim Zemer et al., 2016). After systemic administration, 60 to 70 % of MTX is recovered unchanged in the urine (Evans et al., 1983), whereas 30 % is oxidized by a hepatic metalloflavoprotein, aldehyde oxidase, to 7-hydroxymethotrexate (7OHMTX) (Kitamura et al., 1999). Compared with MTX, the pharmacokinetic variability of 7OHMTX is much less well-defined, and hasn’t been characterized in infants and young children with brain tumors receiving HDMTX. Both MTX and 7OHMTX have limited aqueous solubility, particularly at acid pH, and both compounds may contribute to the formation of nephrolithiasis although the role of 7OHMTX systemic exposures in MTX-associated clinical toxicities has not yet been defined.

In this work we aimed to gain a better understanding of the pharmacokinetics of HDMTX in infants and young children with malignant brain tumors and study the factors influencing delayed MTX excretion and the development of toxicities including the roles of 7OHMTX. Our objectives were to develop a population pharmacokinetic model of MTX and 7OHMTX in infants and young children with brain tumors, to identify the sources of pharmacokinetic variability, and to assess whether MTX and 7OHMTX systemic exposure were related to toxicity. Ultimately, as with our previous analyses, the results of this analysis will be used to refine the recommendations for HDMTX dosing for infants and young children with brain tumors in future clinical studies.

## 2. Patients and methods

### 2.1. Patient population and study design

The pharmacokinetic study was conducted as part of SJYC07 (NCT00602667), a multi-institutional clinical trial evaluating risk-adapted therapy for young children (≤5 years) with primary brain tumors (Robinson et al., 2018). The clinical study was approved by the St. Jude Institutional Review Board and followed ethical principles of the Declaration of Helsinki. Written informed consent was obtained by the patients’ guardians. After tumor resection, patients first received four 28-day cycles of induction therapy including HDMTX and conventional dosage vincristine, cisplatin, and cyclophosphamide.

HDMTX was given on day 1 of each induction cycle as 24-h infusion of 5 g/m^2^ or 2.5 g/m^2^ for very young infants ≤ 31 days at enrollment. A loading dose (i.e., 10 % of total HDMTX) was administered over one hour and the remaining 90 % of HDMTX was administered as a 23-h infusion. Per protocol, all patients received pre-HDMTX hydration and urine pH adjustment. Additionally, patients received leucovorin rescue starting 42 h after HDMTX administration (Panetta et al., 2020). Standard leucovorin regimen was 15 mg/m^2^ IV every 6 h for 5 doses. The leucovorin rescue was adjusted in case of delayed MTX excretion defined as MTX concentration, measured by immunoassay, > 1 μM at 42 h or > 0.1 μM at 66 h. In such cases, additional IV hydration was given and the leucovorin dosage was increased and continued until MTX concentrations fell below 0.03 μM.

### 2.2. Pharmacokinetic sampling and bioanalysis

Pharmacokinetic studies were performed in all patients enrolled at St. Jude during the induction therapy cycles. Blood samples (1–2 mL) were collected pre-infusion and at 6, 23, 42, and 66 h from start of MTX infusion. Additional blood samples were collected every 24 h for patients with any evidence of a fluid collection, clinical toxicity, or delayed drug clearance, until MTX concentrations were undetectable. Each sample was collected in K_2_EDTA tube and centrifuged at room temperature at 7000 g for 10 min within 1-h of blood draw to collect plasma, which was stored at −20 °C until further analysis.

Plasma was assayed for MTX using the Abbott TDx clinical instrument, which uses a fluorescence polarization immunoassay technology (Panetta et al., 2020). The lower limit of quantification (LLOQ) for MTX was 0.03 μM. Plasma was also assayed for MTX and 7OHMTX using a validated LC-MS/MS assay (Roberts et al., 2016). For this method, the LLOQ values were 0.0022 μM for MTX and 0.0085 μM for 7OHMTX. Agreement between TDx and LC-MS/MS assays to measure MTX plasma concentrations was estimated using Bland-Altman difference plots in Prism (version 9.3.1, GraphPad Software, San Diego, California USA). Samples below the LLOQ were excluded from the analysis.

### 2.3. Genotyping assays

In consenting patients, whole blood was collected for isolation of germline DNA prior to beginning therapy. DNA was extracted with a Gentra Puregene Blood Kit (#158,389, Qiagen), following manufacturer’s instructions, and quantified using Nanodrop 2000 Spectrophotometer (Thermo Fisher Scientific). Genome-wide genotyping was performed using the Illumina Infinium Omni2.5Exome-8 BeadChip (Illumina Inc., San Diego, CA). Single nucleotide polymorphisms (SNP) were selected from genes involved in MTX/7OHMTX disposition and transport, according to those previously reported (see Supplementary Table S1). Each SNP was coded in one of three genetic inheritance models (i.e., additive, dominant, and recessive).

### 2.4. Intracranial fluid collections assessments

The volumes (mL) of postoperative intracranial fluid collections were measured in patients after tumor resection and before the start of each induction therapy using magnetic resonance imaging as previously described (Wright et al., 2015). Wilcoxon-Mann-Whitney (WMW) tests were used to compare differences between patients with and without intracranial fluid collections. Random coefficient models were used to explore potential changes of intracranial fluid collections over time.

### 2.5. Population pharmacokinetic and covariate analysis

MTX and 7OHMTX plasma concentrations measured with the LC-MS/MS method were simultaneously analyzed using population-based pharmacokinetic modeling in Monolix (version 2020R1. Antony, France: Lixoft SAS, 2020. http://lixoft.com/products/monolix/). Model parameters were estimated using the Stochastic Approximation Expectation Maximization algorithm. Inter-individual variability (IIV) and inter-occasion variability (IOV) terms were assumed log-normally distributed and were implemented on fixed-effect parameters with an exponential model. Additive and/or proportional error models were used to describe the residual unexplained variabilities. Residual error terms were assumed normally distributed. MTX dosage (i.e., MTX dosage normalized to patient body surface area (BSA)) was used as model input. All data below the LLOQ were censored according to the Beal method M3 (Beal, 2001). Based on previously published MTX models, a two-compartment model was used to describe MTX plasma disposition. For 7OHMTX, one and two-compartment model structures were tested. To avoid parameter identifiability issues, the volume of the central compartment for 7OHMTX was fixed to the central volume of MTX. This approach allowed for estimating two central clearance parameters for MTX (i.e., non-metabolic and metabolic clearances).

Model selection was based on goodness-of-fit plots and changes in the objective function value (Nguyen et al., 2017). The precision of parameter estimates was evaluated using relative standard errors RSE% and 90^th^ confidence intervals from non-parametric bootstraps (*N* = 100). Prediction-corrected visual predictive checks (pcVPC) were used for internal model validation (Bergstrand et al., 2011). Five hundred replicates of the original dataset were simulated using the final model parameters. For both MTX and 7OHMTX, observed concentration-time data were overlaid on the 5^th^, 50^th^, and 95^th^ percentiles of the model simulations for visual assessment. Shrinkage values, based on the estimated random effect variances, were acceptable if < 50 % (Savic and Karlsson, 2009).

A first covariate analysis was performed to explore potential associations between model parameters and the following patient demographics and laboratory values: sex, age, BSA, actual bodyweight, height, intracranial fluid volumes, albumin, alkaline phosphatase, alanine amino-transferase, aspartate amino-transferase, total bilirubin, serum creatinine, and estimated glomerular filtration rate (eGFR) using the full equation established previously (Millisor et al., 2017). For each occasion, the covariate baseline values (i.e., before dose administration) were considered. The changes in eGFR values throughout treatment cycle were also considered. A second covariate analysis explored the influence of SNPs on drug disposition. SNPs were tested if at least 10 % of patients exhibited at least one variant allele. The three genetic inheritance models (i.e., additive, dominant, and recessive) were explored if each class accounted for at least 5 % of patients. Continuous covariates were evaluated as a power model centered on the median covariate value. Categorical covariates were modeled using an exponential model. Covariates were selected with a forward/backward stepwise approach, with criteria *P* values of 0.05 and 0.01 for the forward and backward steps, respectively (Joerger, 2012). The model predictive performance was re-evaluated after the covariate analysis, as described above.

Model-derived drug exposures were defined as the area under the curves (AUC) and were computed using the integrals of the concentration-time curve for both MTX and 7OHMTX.

### 2.6. Characterization of methotrexate delayed excretion

The characteristics of patients who experienced protocol defined delayed MTX excretion were explored compared to patients without delayed excretion. The main patient demographics, laboratory values, and intracranial fluid collection measurements were considered.

Potential differences in the proportion of patients exhibiting delayed drug excretion across induction cycles were analyzed using Fisher exact test. Random intercept logistic regression models were built to explore associations between delayed MTX excretion (Y/N) and patient variables as described above to consider potential differences among cycles. A significance threshold of 0.05 was used without adjusting for multiplicity. This analysis was performed using R version 4.0.5 in RStudio version 1.4.

### 2.7. Exposure-toxicity analysis

In this analysis, MTX-related toxicity was expressed as a binomial variable (i.e., 0 or 1). Toxicities were included in the analysis if they were attributed to MTX therapy, Grade 3 or above according to the Cancer Therapy Evaluation Program, Common Terminology Criteria for Adverse Events, version 3.0, and occurred after start of MTX therapy but before administration of the next chemotherapeutic agent. Two pharmacokinetic variables were explored for association with toxicities: model-derived 7OHMTX AUC_0-∞_ and the sum of model-derived MTX + 7OHMTX AUC_0-∞_. Exposure-toxicity associations were investigated using random intercept logistic regression models which allowed for subject-specific intercepts to consider the different induction cycles (i.e., multiple cycles within a subject), using SAS (SAS Institute, Cary, NC, Windows version 9.4). A significance threshold of 0.05 was used without adjusting for multiplicity.

## 3. Results

### 3.1. Data summary

MTX and 7OHMTX plasma concentration-time data were obtained in 513 induction cycles from 142 unique patients. Among this cohort, 40 patients were less than 1 year of age and 2 patients were less than 1 month of age prior to starting MTX therapy. All patient characteristics are summarized in Table 1. Of the 2142 plasma samples analyzed by both methods, only 17 MTX and 20 7OHMTX samples were below the LLOQ for the LC-MS/MS method. Genotyping assays were performed in 117 patients. A total of 22 SNPs from 9 genes involved in MTX and folate transport and metabolism were extracted (Supplementary Table S1).

The presence of any intracranial fluid collection was assessed in 131 patients and was identified in 87 patients with fluid volumes ranging from 120.1 to 1551 mL/m^2^ (median 392.3 mL/m^2^) across all induction cycles. The volumes of fluid collection significantly decreased across cycles (*P* = 0.0022) but were similar across patient age range (Supplementary Fig. S1A–B). Patients with abnormal fluid collections were younger and had lower BSA values but similar eGFR values compared to patients with no abnormal fluid collections across cycles (Supplementary Fig. S1C–E).

### 3.2. Comparison of methotrexate plasma assays

The comparison of MTX plasma concentrations by TDx and by LC-MS/MS assays is shown in Supplementary Fig. S2. A total of 2142 MTX plasma concentrations were included in this analysis. A good correlation was observed between the two bioanalytical assays, despite a positive bias for the TDx assay at lower concentrations (Supplementary Fig. S2A). The Bland-Altman plots showed a slight positive bias of 7 % for concentrations above 2 μM (Supplementary Fig. S2B) and a higher positive bias of 24.5 % for concentrations below 2 μM (Supplementary Fig. S2C).

### 3.3. Pharmacokinetic modeling and covariate analysis

MTX and 7OHMTX plasma concentration-time profiles were simultaneously described using two-compartment models (Supplementary Fig. S3). MTX elimination was modeled with a linear non-metabolic clearance (CL) and metabolic clearance (CL_MET_) which described the formation of 7OHMTX. 7OHMTX elimination was modeled using a linear clearance (CL_7OH_). The population pharmacokinetic parameters and the diagnostic plots related to the base model (i.e., with no covariate) are reported in Table 2 and Supplementary Fig. S4. Estimation of correlation between random-effects parameters was not supported by the model. Shrinkage values were all below 40 %.

**Fig. 1.**
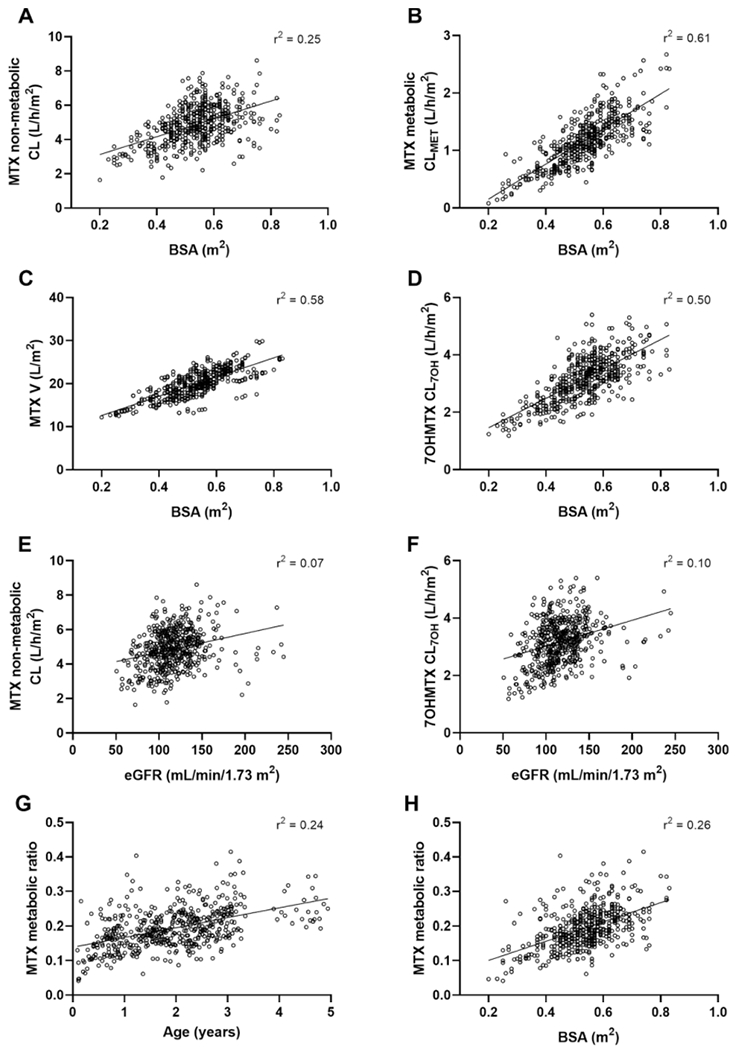
Pharmacokinetic covariate associations with body surface area (BSA) and estimated glomerular filtration rate (eGFR). A) Scatter plots of individual methotrexate (MTX) non-metabolic clearance CL vs BSA. B) Scatter plots of individual MTX metabolic clearance CL_MET_ vs BSA. C) Scatter plots of individual MTX volume V vs BSA. D) Scatter plots of individual 7-hydroxymethotrexate (7OHMTX) clearance CL_7OH_ vs BSA. E) Scatter plots of individual MTX CL vs eGFR. F) Scatter plots of individual 7-hydroxymethotrexate (7OHMTX) clearance CL_7OH_ vs eGFR. Individual MTX metabolic ratio values (ratio CL_MET_ over the sum of CL and CL_MET_) vs age (G) and BSA (H). R^2^ values correspond to Spearman linear correlations.

The first covariate analysis performed on all patients showed the significant influence of baseline BSA and eGFR values on both MTX and 7OHMTX plasma disposition. Patient BSA was positively associated with MTX CL, CL_MET_, volume (V), and CL_7OH_ even though these parameters were already BSA-normalized as MTX dosage (e.g., mg/m^2^) was used as a model input (Fig. 1A–D). Implementation of a BSA effect explained 17.9, 14.3, 24.8, and 20.7 % of the initial IIV estimated on CL, V, CL_MET_, and CL_7OH_, respectively. Patient eGFR was positively associated with CL and CL_7OH_, explaining an additional ~5 % of the variability estimated on these parameters (Fig. 1E-F). Overall, BSA was slightly correlated with eGFR but highly correlated with age, which did not explain any further pharmacokinetic variability (Supplementary Fig. S5). As a result, MTX metabolic ratios, calculated as CL_MET_ divided by the total MTX clearance (i.e., CL + CL_MET_), increased with increasing BSA and age (Fig. 1G and H).

**Fig. 2.**
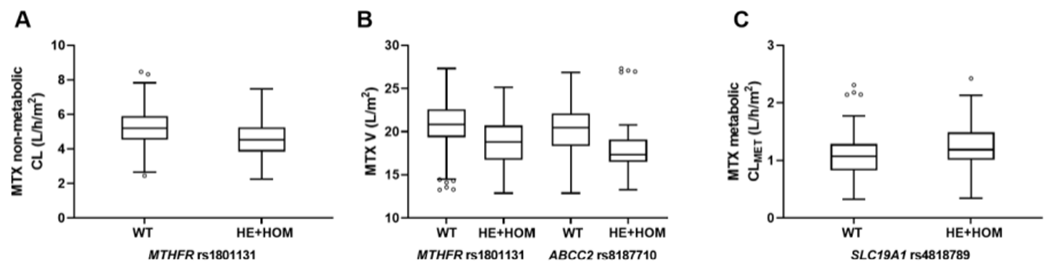
Pharmacokinetic covariate associations with genetic polymorphisms. A) Boxplots of individual methotrexate (MTX) non-metabolic clearance CL vs *MTHFR* rs1801131 categorized as wild-type (WT) or combined heterozygous and homozygous mutant (HE+HOM). B) Boxplots of individual MTX volume V vs *MTHFR* rs1801131 and *ABCC2* rs8187710. C) Boxplots of individual MTX metabolic clearance CLMET vs *SLC19A1* rs4818789. All covariate associations had a p value of < 0.05.

The pharmacogenetic covariate analysis was performed in 117 patients and revealed the association between three SNPs and the pharmacokinetic parameters when implemented as a dominant model (Fig. 2A–C). Patients with at least one variant allele of *MTHFR* rs1801131 exhibited lower MTX CL and V values. Patients with at least one variant allele of *ABCC2* rs8187710 also exhibited lower V. Lastly, patients with at least one variant allele of *SLC19A1* rs4818789 had higher CL_MET_. The inclusion of these SNP effects explained 6.6, 13.2, and 6.6 % of CL, V, and CL_MET_ variabilities initially estimated in this reduced population.

Intracranial fluid collection volume was evaluated as both a categorical (presence/absence) and a continuous covariate but did not show any significant association with MTX and 7OHMTX pharmacokinetic parameters (Supplementary Fig. S6).

The final equations describing the associations between covariates and pharmacokinetic parameters are reported below:

$$CL_{i}=CL_{pop}\times {\left(\frac{BSA_{i}}{BSA_{pop}}\right)}^{\theta_{BSA,CL}}\times {\left(\frac{eGFR_{i}}{eGFR_{pop}}\right)}^{\theta_{eGFR,CL}}\times {\left(\theta_{MTHFR,CL}\right)}^{MTHFR}$$

$$V_{i}=V_{pop}\times {\left(\frac{BSA_{i}}{BSA_{pop}}\right)}^{\theta_{BSA,V}}\times {\left(\theta_{MTHFR,V}\right)}^{MTHFR}\times {\left(\theta_{ABCC2}\right)}^{ABCC2}$$

$$CL_{MET,i}=CL_{MET,pop}\times {\left(\frac{BSA_{i}}{BSA_{pop}}\right)}^{\theta_{BSA,CLmet}}\times {\left(\theta_{SLC}\right)}^{SLC19A1}$$

$$CL_{7OH,i}=CL_{7OH,pop}\times {\left(\frac{BSA_{i}}{BSA_{pop}}\right)}^{\theta_{BSA,CL7oh}}\times {\left(\frac{eGFR_{i}}{eGFR_{pop}}\right)}^{\theta_{eGFR,\;CL7oh}}$$

where each *P_i_* is the individual parameter and *P_pop_* is the mean population estimate. MTHFR, ABCC2, and SLC19A1 refer to the genotype (0 for wild-type, 1 for heterozygous or homozygous mutant). The Θ terms represent the estimated covariate coefficients.

The parameter estimates for the two final covariate models (i.e., BSA/eGFR and BSA/eGFR/SNP) are reported in Table 2. All the parameters were estimated with good precision (RSE% <50 %). The central tendency and the variability of MTX and 7OHMTX plasma concentrations were adequately predicted by the two covariate models. The pcVPC performed for the BSA/eGFR covariate model are shown in Supplementary Fig. S7.

**Fig. 3.**
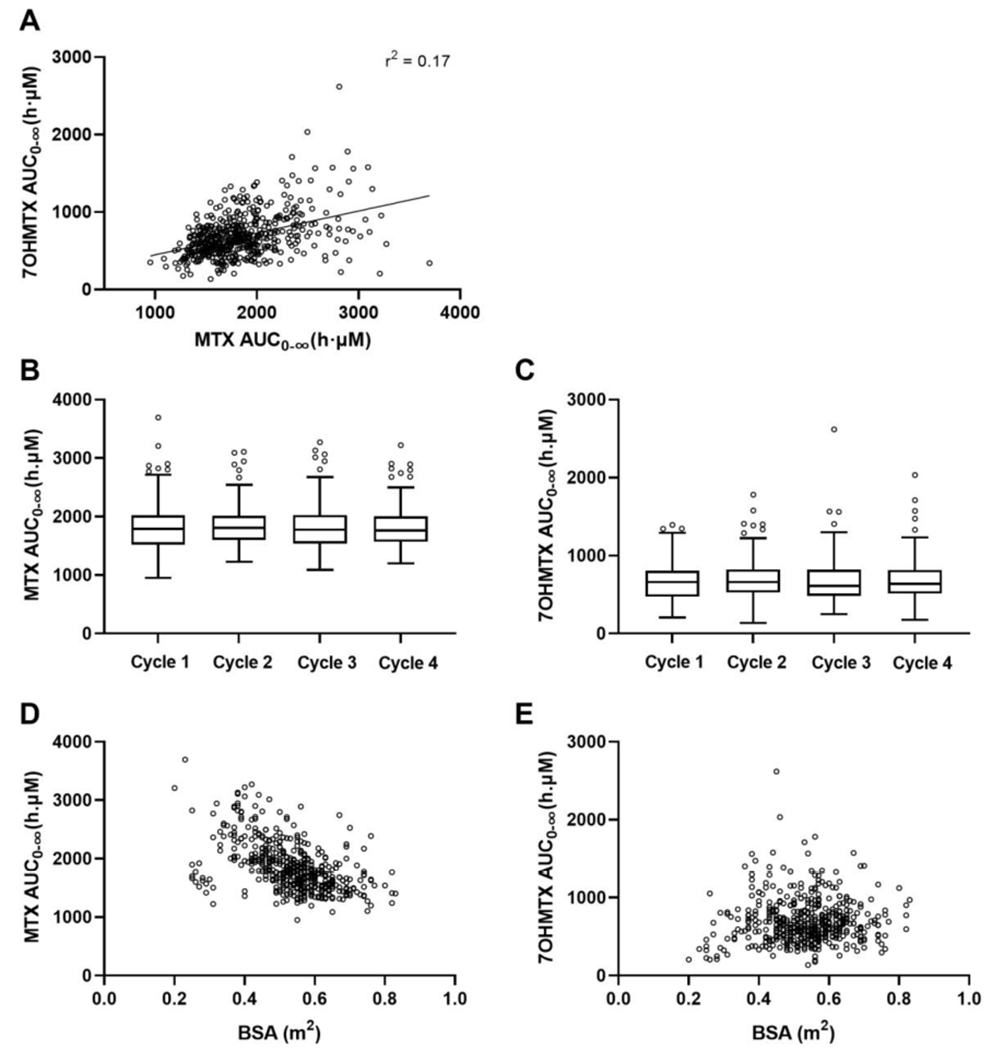
Methotrexate (MTX) and 7-hydroxymethotrexate (7OHMTX) exposures. A) Correlations between MTX and 7OHMTX AUC_0-∞_. Boxplots of individual MTX (B) and 7OHMTX (C) AUC_0-∞_ per induction therapy cycle. Scatter plots of individual MTX (D) and 7OHMTX (E) AUC_0-∞_ vs BSA.

Individual plasma AUC_0-∞_ values were derived from the final BSA/eGFR model. Mean values were 1851.5 and 689.1 h⋅μM for MTX and 7OHMTX, respectively. Parent drug and metabolite AUC_0-∞_ values were moderately correlated (Fig. 3A) and did not significantly change across cycles (Fig. 3B). Patient BSA was statistically negatively correlated with MTX AUC_0-∞_ but not with 7OHMTX AUC_0-∞_ as shown in Fig. 3D and E.

### 3.4. Methotrexate delayed excretion

Considering the positive bias of 1.24-fold we estimated between TDx and LC-MS/MS assays for low MTX concentrations, delayed MTX excretion was redefined as LC-MS/MS measured concentrations above 0.8 and 0.08 μM at 42 h and 66 h, respectively.

Individual predicted MTX concentrations using the BSA/eGFR model were used to explore delayed excretions occurring in the studied population. The numbers of patients experiencing delayed excretion were reported in Table 3A. Most of patients with a delayed excretion at 42 h still exhibited delayed excretion at 66 h. However, about half of the patients with delayed excretion at 66 h did not show one at 42 h. No significant difference was observed in the proportion of patients with delayed excretion between cycles at both 42 h (*P* = 0.86) and 66 h (*P* = 0.76). This statement remained true in all subsequent analyses.

Several characteristics (e.g., age, BSA, eGFR, and intracranial fluid volumes) were compared between patients with and without delayed excretion (Table 3B and Supplementary Table S3). Using random intercept logistic regression univariable models, we found that patients with delayed MTX excretion at 42 h were younger (*P* = 0.0001), had lower BSA (*P* = 7.15e-06), but similar eGFR (*P* = 0.703), and intracranial fluid collections in terms of proportions (i.e., presence/absence, *P* = 0.319) and actual volumes (*P* = 0.318) compared to patients with no delayed excretion. Similarly, patients with delayed MTX excretion at 66 h had lower BSA (*P* = 0.0037), but similar age (*P* = 0.24), eGFR (*P* = 0.43), and intracranial fluid collections in terms of proportions (i.e., presence/absence, *P* = 0.072) and actual volumes (*P* = 0.084) compared to patients with no delayed excretion.

### 3.5. Exposure-toxicity analysis

MTX-related toxicities were reported for 24 unique patients with a total of 30 events: 28 Grade 3 and 2 Grade 4 toxicity events. Most of these toxicities (> 80 %) were defined as possibly related to MTX therapy and were gastrointestinal toxicities including ulcerative stomatitis and severe diarrhea. The details of the adverse events are reported in Supplementary Table S2.

No significant associations were found between the two pharmacokinetic variables and the toxicity events observed in this population. MTX-related toxicity incidence did not vary across induction cycles (*P* = 0.48) and was not associated with 7OHMTX AUC (*P* = 0.44) or the sum of 7OHMTX and MTX AUC (*P* = 0.92).

## 4. Discussion

A population pharmacokinetic analysis was conducted to describe MTX and 7OHMTX concentration-time data observed after HDMTX therapy in a large population of infants and young children with malignant brain tumors. The model best describing MTX and 7OHMTX concentration-time data consisted of two-compartment models for both MTX and 7OHMTX including terms to describe MTX elimination and 7OHMTX formation. Covariate analysis showed that baseline BSA explained variability in CL, V, CL_MET_, and CL_7OH_, and eGFR explained variability in CL and CL_7OH_. A pharmacogenetic covariate analysis identified several SNPs in genes involved in MTX/7OHMTX disposition and transport having influence including *MTHFR* (rs1801131), *ABCC2* (rs8187710), and *SLC19A1* (rs4818789). Lastly, no relationship was observed between MTX, 7OHMTX, or the sum of MTX and 7OHMTX systemic exposure and toxicity.

Our previous MTX population analysis used concentrations that were analyzed with an immunoassay method (Pesce and Bodourian, 1986), but to obtain 7OHMTX concentrations, we used a specific, sensitive, and robust LC-MS/MS method (Roberts et al., 2016). We compared MTX plasma concentration determined by immunoassay and LC-MS/MS and, similar to other investigators, found a good correlation between the methods (Bouquié et al., 2016; Günther et al., 2016). We did find a positive bias for the immunoassay at lower concentrations, especially at concentrations lower 2 μM. The positive bias observed could be attributed to cross-reactivity to other molecules (e.g., 7OHMTX), variations in matrix effects (e.g., slightly lipemic or hemolyzed specimens), or differences in detection limits.

Of the published population pharmacokinetic analyses of MTX and 7OHMTX, this is the first solely in infants and young children with malignant brain tumors. We used two-compartment models for MTX and 7OHMTX to simultaneously model our results. This is compared with other investigators (Bore et al., 1987; Joerger et al., 2006) who used similar structural models to describe MTX and 7OHMTX plasma concentration-time data obtained in adults. However, considering the different model parameterizations that were made regarding 7OHMTX, no direct comparisons could be made with our 7OHMTX pharmacokinetic parameters.

Since 1996, over 40 population pharmacokinetic studies of MTX have been published, and of these studies, 19 were conducted in pediatric populations (summarized in Supplementary Table S4). None of these studies included 7OHMTX concentration-time data, four studies were conducted in infants and young children (i.e., < 4.7 y), and only two were performed in children with malignant brain tumors. In 14 of these 19 studies, some measure of body size (body weight or BSA) was identified as a significant covariate of MTX pharmacokinetic parameters. In one of the remaining five studies where a measure of body size was not a significant covariate, creatinine clearance was calculated using the Schwartz formula (Schwartz et al., 1976) which includes body length as an indirect measure of body size. Even though BSA-normalized MTX dosages (e.g., mg/m^2^) were used as model inputs, our covariate analysis showed that a patient’s baseline BSA was positively associated with CL, V, CL_MET_, and CL_7OH_. Our BSA exponent estimate of 0.57 for CL was similar to that of Hui and colleagues (Hui et al., 2019) of 0.72 and Panetta and colleagues of 0.70 (Panetta et al., 2020).

In ten of the published pediatric MTX population pharmacokinetic studies referred to above (Supplementary Table S4), some measure of renal function was associated with MTX systemic clearance. Of those ten, five used serum creatinine and five used a serum creatinine based equation to estimate creatinine clearance (i.e., eGFR) and found that eGFR was positively associated with CL (Rühs et al., 2012). Additionally, we found that eGFR was positively associated with CL_7OH_. Considering that MTX is 80 to 90 % eliminated in the urine, renal function was expected to be a significant covariate on MTX disposition. However, it was surprising that only ~5 % of the variability was accounted for in our model by renal function (i.e., eGFR).

The pharmacogenetic covariate analysis, which consisted of 22 SNPs tested in 9 genes, identified 3 SNPs as significant covariates explaining a modest 6 to 13 % of the estimated interpatient variability. The *MTHFR* rs1801131 genotype was associated with a decrease in both MTX clearance and volume consistent with several previous observations (Faganel Kotnik et al., 2011; Imanishi et al., 2007; Müller et al., 2008). It is not entirely clear by what mechanism this intracellular enzyme alters systemic drug disposition. A significant influence of *ABCC2* rs8187710 genotype on volume of distribution was also observed. ABCC2 is expressed on the canalicular membrane of hepatocytes and on the apical membrane of renal proximal tubules suggesting a possible involvement in the excretion of xenobiotics from the body. This has been observed for other ABCC2 genotypes (Rau et al., 2006). We also found that *SCL19A1* rs4818789 genotype was associated with the formation of 7OHMTX (i.e., CL_MET_). Although the impact of these SNPs was statistically significant, hence included in our model, they were they were not considered clinically relevant enough to be considered for proposing dosing adjustments at this stage.

Previously, the association of intracranial post-resection fluid collections on plasma MTX disposition had been analyzed during a single course of HDMTX in infants and young children with brain tumors. The results of that analysis showed that these patients were more likely to have delayed plasma MTX excretion (Wright et al., 2015). The primary limitation to this study was that patients were only evaluated during one course of HDMTX; thus, clinicians were not provided with recommendations for how to follow patients after multiple HDMTX courses. In the present study, we used a similar approach to evaluate intracranial post-resection fluid collections in patients after tumor resection before the start of each induction HDMTX. As a covariate, the intracranial fluid collection was not significantly associated with to MTX or 7OHMTX disposition, which differed from our previous analysis. This could be attributed to resolution in the fluid collection with additional courses of MTX therapy, developmental changes in renal function improving MTX renal clearance, and the larger number of observations in the present study. We further evaluated the characteristics of patients experiencing delayed MTX excretion compared to patients with no delayed excretion but did not find any other risk factor than measures of age and BSA.

In our previous analysis, we showed that MTX systemic exposure was not associated with grade 3 or 4 adverse events attributable to HDMTX therapy in infants and young children with brain tumors (Panetta et al., 2020). Here, we focused on the role of 7OHMTX in the observed toxicities, but again, no associations were found. It is important to note that, overall, therapy for this clinical trial (i.e., SJYC07; NCT00602667) was very well tolerated with low toxicity (e.g., 30 adverse events in 513 MTX courses; See Supplementary Table S2) and without any grade 3 or 4 nephrotoxicity reported (Robinson et al., 2018). This low observed toxicity is likely due to the aggressive clinical management and leucovorin rescue these patients received. Thus, our lack of association between MTX and 7OHMTX systemic exposure and toxicity events should not be necessarily interpreted as an absence of exposure-toxicity relationship for these compounds as the low number of toxic events hampered our analysis.

The present study had several limitations. First, we didn’t include covariates in our analysis which may have affected MTX or 7OHMTX clearance including hydration, urine pH, leucovorin dosing, concomitant drugs, or MTX/7OHMTX urinary concentrations. Second, the number of patients available with MTX and 7OHMTX concentration-time data were limited and no external validation dataset was available. Lastly, the low observed toxicity rate of our clinical study didn’t allow for a thorough exploration of drug exposure-toxicity associations although this outcome was at the benefit of our patients.

## 5. Conclusion

This was the first population pharmacokinetic study evaluating MTX and 7OHMTX disposition in infants and young children with brain tumors. Significant covariates associated with MTX and 7OHMTX disposition identified in our analysis included baseline patient BSA and eGFR. The genotype analysis only showed minor to modest impact of several SNPs including *MTHFR* rs1801131, *ABCC2* rs8187710, and *SLC19A1* rs4818789. No further factors were found to be predictive of a delayed MTX excretion. HDMTX can be safely administered to infants and young children, even with post-operative intracranial fluid resections, with careful monitoring. We continue to recommend aggressive clinical management and leucovorin rescue for these patients to guide HDMTX therapy and maintain low toxicity rates.

## Supplementary Material

1

## Figures and Tables

**Table 1:** Characteristics of patient population for pharmacokinetic and pharmacogenetic analyses.

Baseline characteristics	Pharmacokinetic analysis	Pharmacogenetic analysis
Total number of patients	142	117
*Induction therapy cycle (%)		
Cycle 1	134 (94.4)	109 (93.2)
Cycle 2	134 (94.4)	110 (94.0)
Cycle 3	124 (87.3)	103 (88.0)
Cycle 4	121 (85.8)	102 (87.2)
Male / Female, n (%)	77 (54.2) / 65 (45.8)	61 (52.1) / 56 (47.9)
Age (years)	1.91 (0.09–4.94)	1.97 (0.09–4.94)
Total body weight (kg)	11.8 (2.8–21.2)	11.8 (3.7–21.2)
Body surface area (m^2^)	0.54 (0.2–0.83)	0.55 (0.25–0.83)
Albumin (U/L)	4.1 (2.8–5.2)	4.1 (2.8–4.8)
Alkaline phosphatase (U/L)	154 (62–541)	153 (62–541)
Alanine aminotransferase (U/L)	18 (5–162)	18 (5.0–105)
Aspartate aminotransferase (U/L)	29 (9.0–186)	28 (9.0–117)
Serum creatinine (mg/dL)	0.2 (0.1–0.45)	0.2 (0.1–0.45)
estimated GFR (mL/min/1.73 m^2^)	114 (50.8–244.8)	114.3 (56.4–244.8)
Total bilirubin (mg/dL)	0.2 (0.1–2.6)	0.2 (0.1–0.8)

Data represented as median (range) or frequency (%) for continuous or categorical characteristics.

* Induction therapy cycle during which pharmacokinetic studies were performed for methotrexate

GFR –glomerular filtration rate.

**Table 2:** Parameter estimates for the base model, BSA and eGFR effects model, and BSA, eGFR, and SNP effects model.

	Parameter estimates (RSE%) [90th CI]		
Parameter (Unit)	Base model (*N* = 142)	BSA, eGFR model (*N* = 142)	BSA, eGFR, SNP model (*N* = 117)
Population parameters			
MTX non-metabolic clearance CL (L/h/m^2^)	4.6 (2.1) [4.S–4.8]	4.7 (1.7) [4.6–4.9]	4.9 (2.5) [4.8–5.1]
BSA effect, θ_BSA,CL_		0.57 (13.1) [0.38–0.69]	0.43 (19.5) [0.30–0.56]
eGFR effect, θ_eGFR,CL_		0.11 (42.6) [0.02–0.19]	0.13 (36.2) [0.040–0.20]
*MTHFR* (rs1801131) variant, θ_MTHFR,CL_			−0.083 (43.1) [−0.13- −0.029]
MTX volume, V (L/m^2^)	19.7 (1.4) [18.9–20.0]	20.1 (1.2) [19.5–20.9]	20.8 (1.8) [20.3–21.8]
BSA effect on V, θ_BSA,V_		0.57 (9.2) [0.44–0.69]	0.46 (12.7) [0.31–0.58]
*MTHFR* (rs1801131) variant, θ_MTHFR,V_			−0.076 (32.3) [−0.12- −0.026]
*ABCC2* (rs8187710) variant, θ_ABCC2_			−0.10 (34.9) [−0.21- −0.013]
MTX peripheral clearance, Q (L/h/m^2^)	0.047 (4.9) [0.044–0.057]	0.047 (5.1) [0.042–0.054]	0.048 (5.6) [0.043–0.055]
MTX peripheral volume, V_p_ (L/m^2^)	1.5 (5.9) [1.3–1.7]	1.5 (6.0) [1.4–1.7]	1.7 (6.8) [1.3–1.8]
MTX metabolic clearance, CL_MET_	1.1 (3.2) [0.99–1.2]	1.2 (2.6) [1.1–1.25]	1.1 (3.4) [0.99–1.2]
BSA effect, θ_BSA,CLmet_		1.7 (6.5) [1.4–2.2]	1.2 (9.9) [0.92–1.6]
*SLC19A1* (rs4818789) variant, θ_SLC_			0.099 (49.1) [0.041–0.18]
7OHMTX clearance, CL_7OH_ (L/h/m^2^)	3.0 (2.0) [2.8–3.2]	3.1 (1.7) [3.0–3.3]	3.0 (1.8) [2.9–3.2]
BSA effect, θ_BSA,CL7oh_		0.84 (8.7) [0.67–1.1]	0.61 (13.4) [0.48–0.87]
eGFR effect, θ_eGFR,CL7oh_		0.16 (34.3) [0.07–0.30]	0.18 (33.1) [0.082–0.30]
7OHMTX peripheral clearance, Q_7OH_ (L/h/m^2^)	0.38 (5.9) [0.32–0.45]	0.40 (6.1) [0.32–0.44]	0.37 (5.9) [0.30–0.43]
7OHMTX peripheral volume, V_p,7OH_ (L/m^2^)	10.5 (7.2) [8.9–12.3]	10.2 (7.2) [8.9–12.4]	9.2 (7.4) [8.4–11.6]

Inter-individual variability (IIV)			

IIV–CL	0.23 (7.2) [0.20–0.26]	0.18 (7.7) [0.16–0.21]	0.17 (8.9) [0.14–0.20]
IIV–V	0.14 (8.7) [0.11–0.17]	0.12 (8.4) [0.10–0.14]	0.11 (10.2) [0.075–0.13]
IIV–Q	0.45 (10.2) [0.38–0.56]	0.48 (9.5) [0.38–0.54]	0.47 (10.3) [0.35–0.55]
IIV–V2	0.62 (7.3) [0.51–0.70]	0.64 (7.2) [0.55–0.74]	0.68 (7.4) [0.56–0.78]
IIV–CL_MET_	0.31 (9.2) [0.24–0.41]	0.23 (9.2) [0.18–0.27]	0.20 (11.6) [0.15–0.24]
IIV–CL_7OH_	0.16 (11.9) [0.11–0.21]	0.11 (15.0) [0.07–0.14]	0.11 (16.1) [0.046–0.13]
IIV–Q_7OH_	0.53 (8.9) [0.42–0.72]	0.55 (8.9) [0.43–0.71]	0.46 (9.7) [0.43–0.67]
IIV–V_p,7OH_	0.70 (8.5) [0.57–81]	0.70 (8.0) [0.64–0.88]	0.69 (8.4) [0.60–0.90]

Inter-occasion variability (IOV)			

IOV–CL	0.12 (5.7) [0.11–0.14]	0.12 (5.3) [0.11–0.13]	0.12 (5.9) [0.10–0.14]
IOV–V	0.051 (14.1) [0.033–0.063]	0.047 (14) [0.032–0.063]	0.052 (13.1) [0.040–0.068]
IOV–Q	0.31 (6.6) [0.25–0.40]	0.33 (6.1) [0.27–0.41]	0.36 (7.3) [0.29–0.42]
IOV–CL_MET_	0.20 (6.5) [0.18–0.23]	0.20 (6.4) [0.17–0.23]	0.20 (7.6) [0.16–0.22]
IOV–CL_7OH_	0.17 (5.1) [0.14–0.19]	0.17 (5.2) [0.14–0.18]	0.17 (5.3) [0.15–0.20]

Proportional residual error			

MTX	0.22 (2.3) [0.20–0.23]	0.21 (2.2) [0.19–0.22]	0.21 (2.3) [0.19–0.22]
7OHMTX	0.25 (1.95) [0.23–0.26]	0.24 (2.0) [0.23–0.26]	0.24 (2.2) [0.22–0.25]

MTX, methotrexate; 7OHMTX, 7-hydroxymethotrexate; RSE, relative standard error; BSA body surface area; eGFR, estimated glomerular filtration rate; SNP, single nucleotide polymorphisms; CI, confidence interval.

Each genetic variant was implemented using a dominant inheritance model: wild-type (reference) versus combined heterozygous and homozygous mutated (variant).

Inter-individual and inter-occasion variabilities are reported as standard deviation.

In both final models, the additive residual error was fixed to 0.001 μM.

90th confidence interval values were obtained from non-parametric bootstrap (*N* = 100).

**Table 3A:** Number of patients with methotrexate delayed excretion (DE).

Cycle	Total patients	DE at 42 h	DE at 66 h	DE at 42 and 66 h
Cycle 1	134	34	57	30
Cycle 2	134	39	58	33
Cycle 3	124	32	55	29
Cycle 4	121	30	59	27

**Table 3B:** Characteristics of patients with methotrexate delayed excretion (DE) during cycle 1.

Variable	Patients with DE at 42 h	Patients with no DE at 42 h	Patients with DE at 66 h	Patients with no DE at 66 h
Age (years)	1.22 ± 1.05	1.98 ± 0.90	1.56 ± 1.05	1.95 ± 0.92
BSA (m^2^)	0.45 ± 0.13	0.56 ± 0.09	0.49 ± 0.12	0.56 ± 0.10
eGFR (mL/min/m^2^)	114.3 ± 35.3	123.1 ± 26.0	125.8 ± 36.3	117.2 ± 20.7
Intracranial fluid collections				
N with / N without	23 / 9	54 / 35	38 / 14	39 / 30
Volumes (mL/m^2^)	466.8 ± 229.2	475.3 ± 245.5	495.6 ± 260.2	450.4 ± 218.1

BSA, body surface area; eGFR estimated glomerular filtration rate.

Data are reported as mean ± standard deviation.

## Data Availability

Data will be made available on request.
