# Supplementary material for: Population pharmacokinetics of methotrexate and 7-hydroxymethotrexate and delayed excretion in infants and young children with brain tumor
Source: Eur J Pharm Sci. Author manuscript; Available in PMC 2024 Feb 5. (PMC10843628; doi:10.1016/j.ejps.2023.106669)
Supplement: 1 [file NIHMS1960113-supplement-1.docx]

**Population pharmacokinetics of methotrexate and 7-hydroxymethotrexate in infants and young children with brain tumors**

**SUPPLEMENTARY MATERIAL**

Corresponding author: Clinton F. Stewart, Pharm.D., Department of Pharmaceutical Sciences, St. Jude Children’s Research Hospital, 262 Danny Thomas Place, Memphis, TN 38105-2794, Telephone: (901) 595-3665; FAX: (901) 525-6869 E-mail: [clinton.stewart@stjude.org](mailto:clinton.stewart@stjude.org)

**CONTENTS:**

| **Page** | **Label** | **Description** |
| --- | --- | --- |
| 2 | Table S1 | Selected genotypic variants evaluated in the population-based pharmacokinetic analysis for methotrexate and its metabolites. |
| 3 | Figure S1 | Boxplots of intracranial fluid collection volumes per induction therapy cycle. |
| 4 | Figure S2 | Bland-Altman plots for MTX plasma concentrations measured by TDx immunoassay (reference method) *vs* LC-MS/MS, for all data (A), data above 2 µM (B), and data below 2 µM (C). |
| 5 | Figure S3 | Pharmacokinetic model structure for MTX and 7OHMTX. |
| 6 | Figure S4 | Goodness-of-fit plots of the pharmacokinetic base model for MTX and 7OHMTX. |
| 7 | Figure S5 | Associations between patient BSA and eGFR or age |
| 8 | Figure S6 | Scatter plots of MTX and 7OHMTX parameters *vs* intracranial fluid collection volumes |
| 9 | Figure S7 | Prediction-corrected visual predictive checks for the final BSA-eGFR model. |
| 10 | Table S2 | MTX-related toxicities |
| 11 | Table S3 | Characteristics of patients with methotrexate delayed excretion (DE) during cycles 2, 3, and 4. |
| 12 | Table S4 | Summary of pediatric MTX population PK studies from 1999 to 2023. |

**Throughout the data supplement, the following abbreviations will be used:**

MTX, methotrexate

7OHMTX, 7-hydroxymethotrexate

## TABLE S1. Selected genotypic variants evaluated in the population-based pharmacokinetic analysis for MTX and 7OHMTX

|  |  | **WT Patients** | | **HE Patients** | | **HOM Patients** | | **Unknown** | |
| --- | --- | --- | --- | --- | --- | --- | --- | --- | --- |
| **SNP** | **Gene** | **n** | **%** | **n** | **%** | **n** | **%** | **n** | **%** |
| rs1045642 | ABCB1 | 36 | 30.8 | 58 | 49.6 | 23 | 19.7 | - | - |
| rs1858923 | ABCB1 | 40 | 34.2 | 51 | 43.6 | 26 | 22.2 | - | - |
| rs2032582 | ABCB1 | 38 | 32.5 | 59 | 50.4 | 20 | 17.1 | - | - |
| rs868755 | ABCB1 | 45 | 38.5 | 57 | 48.7 | 15 | 12.8 | - | - |
| rs2074087 | ABCC1 | 81 | 69.2 | 28 | 23.9 | 8 | 6.8 | - | - |
| rs2239330 | ABCC1 | 65 | 55.6 | 48 | 41.0 | 4 | 3.4 | - | - |
| rs3740066 | ABCC2 | 51 | 43.6 | 49 | 41.9 | 16 | 13.7 | 1 | 0.9 |
| rs2273697 | ABCC2 | 89 | 76.1 | 25 | 21.4 | 3 | 2.6 | - | - |
| rs717620 | ABCC2 | 80 | 68.4 | 32 | 27.4 | 5 | 4.3 | - | - |
| rs8187710 | ABCC2 | 101 | 86.3 | 15 | 12.8 | 1 | 0.9 | - | - |
| rs868853 | ABCC4 | 88 | 75.2 | 26 | 22.4 | 1 | 0.9 | 2 | 1.7 |
| rs9516519 | ABCC4 | 90 | 76.9 | 25 | 21.4 | 2 | 1.7 | - | - |
| rs2231142 | ABCG2 | 93 | 79.5 | 22 | 18.8 | 2 | 1.7 | - | - |
| rs13120400 | ABCG2 | 76 | 65.0 | 33 | 28.2 | 8 | 6.8 | - | - |
| rs2241080 | AOX1 | 62 | 53.0 | 44 | 37.6 | 11 | 9.4 | - | - |
| rs2293528 | AOX1 | 52 | 44.4 | 53 | 45.3 | 12 | 10.3 | - | - |
| rs1801131 | MTHFR | 61 | 52.1 | 48 | 41.0 | 8 | 6.8 | - | - |
| rs1801133 | MTHFR | 64 | 54.7 | 46 | 39.3 | 7 | 6.0 | - | - |
| rs1051266 | SLC19A1 | 32 | 27.4 | 55 | 47.0 | 30 | 25.6 | - | - |
| rs4818789 | SLC19A1 | 67 | 57.3 | 40 | 34.2 | 10 | 8.5 | - | - |
| rs11045879 | SLCO1B1 | 86 | 73.4 | 29 | 24.8 | 1 | 0.9 | 1 | 0.9 |
| rs2306283 | SLCO1B1 | 46 | 39.3 | 47 | 40.2 | 24 | 20.5 | - | - |

WT, wild-type; HE, heterozygous; HOM, homozygous mutant; ABCB1, multidrug resistance protein 1; ABCC (1-2-4), multidrug resistance-associated protein, AOX1, aldehyde oxidase 1; MTHFR, methylenetetrahydrofolate; SLC19A1, solute carrier family 19 member 1; SLCO1B1, solute carrier organic anion transporter family member 1B1.

**Figure S1.** Boxplots of intracranial fluid collection volumes per induction therapy cycles (A). Scatter plots of fluid collection volumes *vs* patient age (B). Boxplots of individual BSA (C), age (D), and eGFR (E) values between patients with abnormal fluid collections (stripes) and patients without (clear). *P* values refer to Wilcoxon-Mann-Whitney tests. All boxplots represent the median, 5, and 95^th^ percentiles of the data.

**
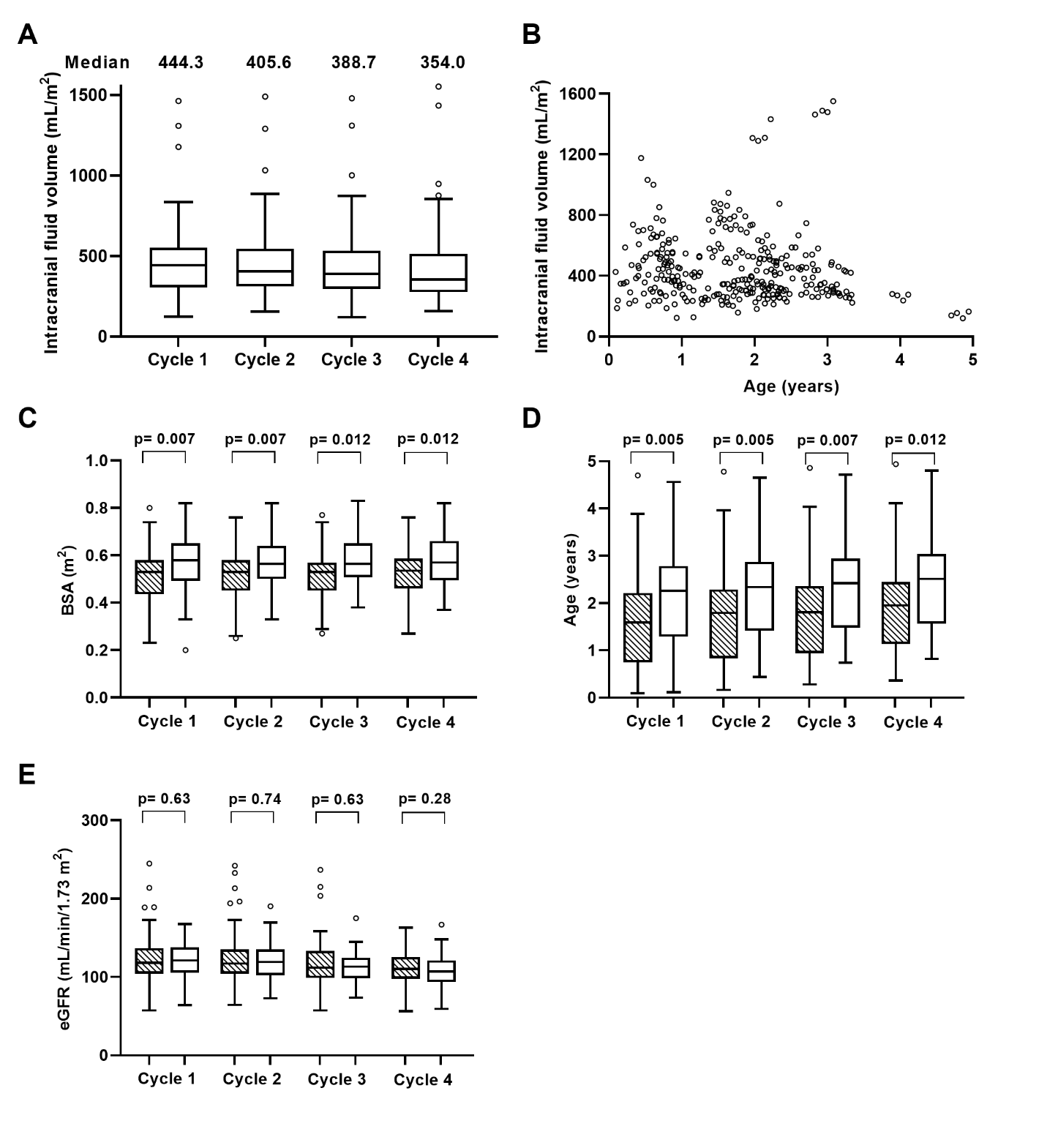
**

**Figure S2.** Bland-Altman plots for MTX plasma concentrations measured by TDx immunoassay (reference method) *vs* LC-MS/MS, for all data (A), data above 2 µM (B), and data below 2 µM (C). The ratio between TDx and LC-MS/MS is plotted against the average of the two measurement methods.


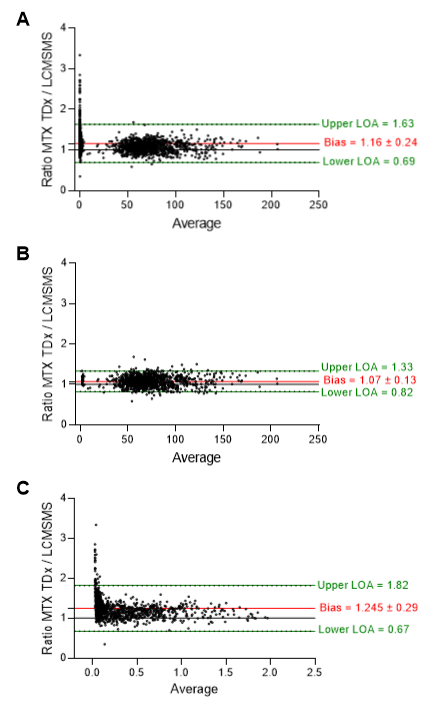


**Figure S3.** Pharmacokinetic model structure for MTX and 7OHMTX


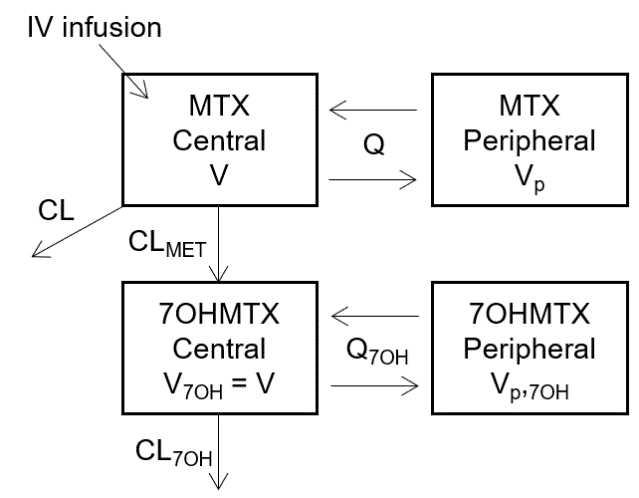


MTX data were described using a two-compartment model (top structure), parameterized with central volume V, central non-metabolic clearance CL, central metabolic clearance CL_MET_, peripheral volume Vp, and clearance Q.

7OHMTX data were described using a two-compartment model (bottom structure), parameterized with central clearance CL_7OH_, peripheral volume V_p,7OH_ and clearance Q_7OH_. 7OHMTX volume of distribution V_7OH_ was set equal to MTX volume V because of parameter identifiability issues.

**Figure S4.** Goodness-of-fit plots of the pharmacokinetic base model for MTX (left) and 7OHMTX (right): observations versus population predictions (A), observations versus individual predictions (B), individual weighted residuals (IWRES) versus time (C), and IWRES versus predictions (D). In panels A and B, solid line represents the identity line. In panels C and F, dotted line is the y = 0 line. In all panels, circles represent individual data and crosses are data below the limit of quantification. The diagnostic plots show a good distribution of the data along the identity line or the y = 0 line for both MTX and 7OHMTX.


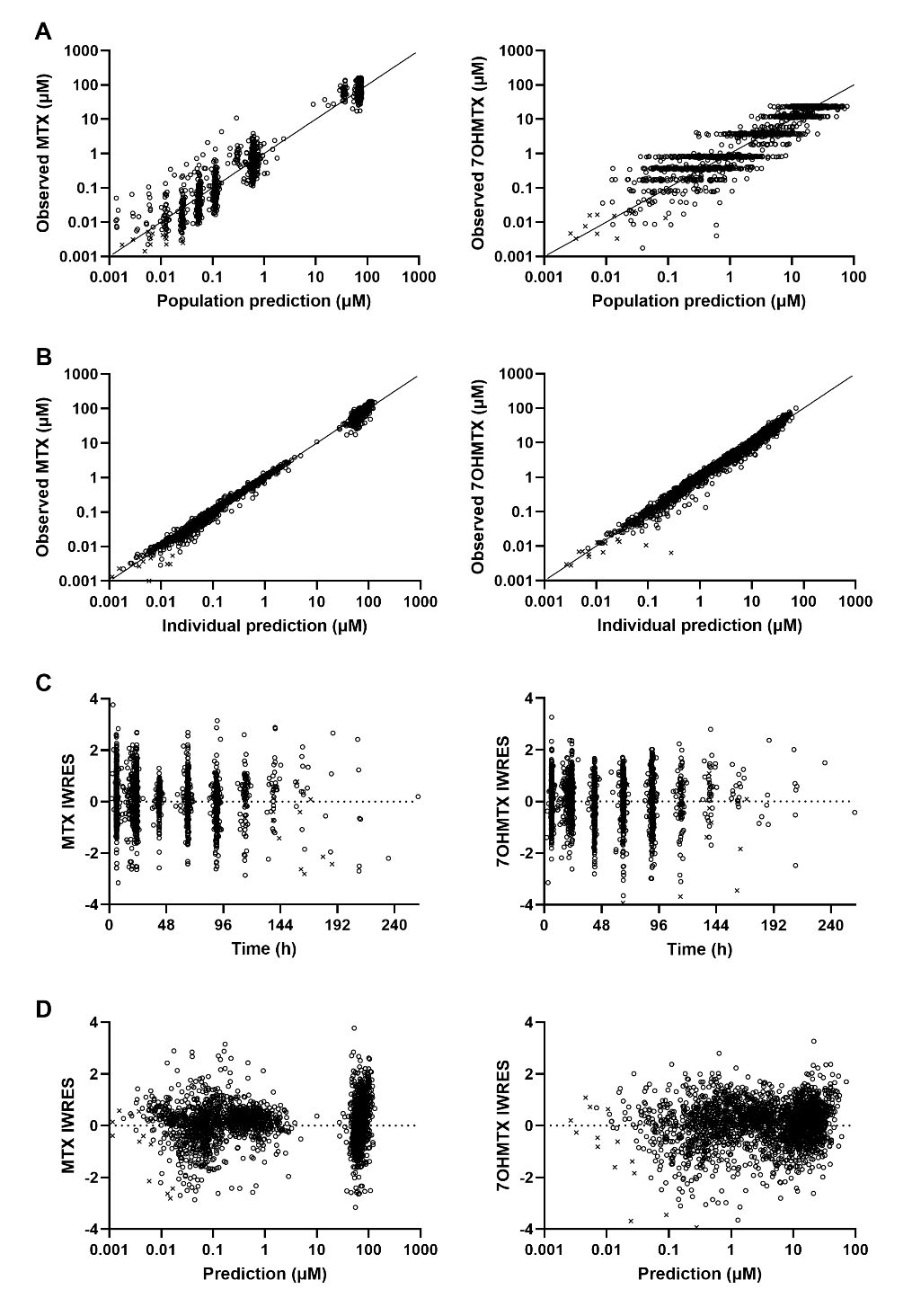


**Figure S5.** Associations between patient eGFR and age or BSA


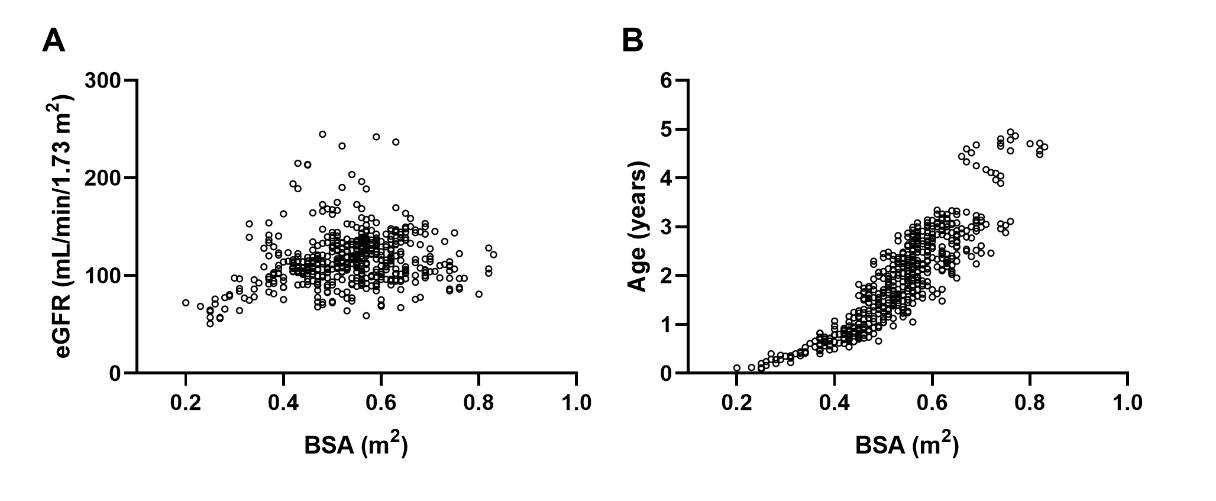


**Figure S6.** Scatter plots of MTX and 7OHMTX pharmacokinetic parameters *vs* intracranial fluid collections volumes.


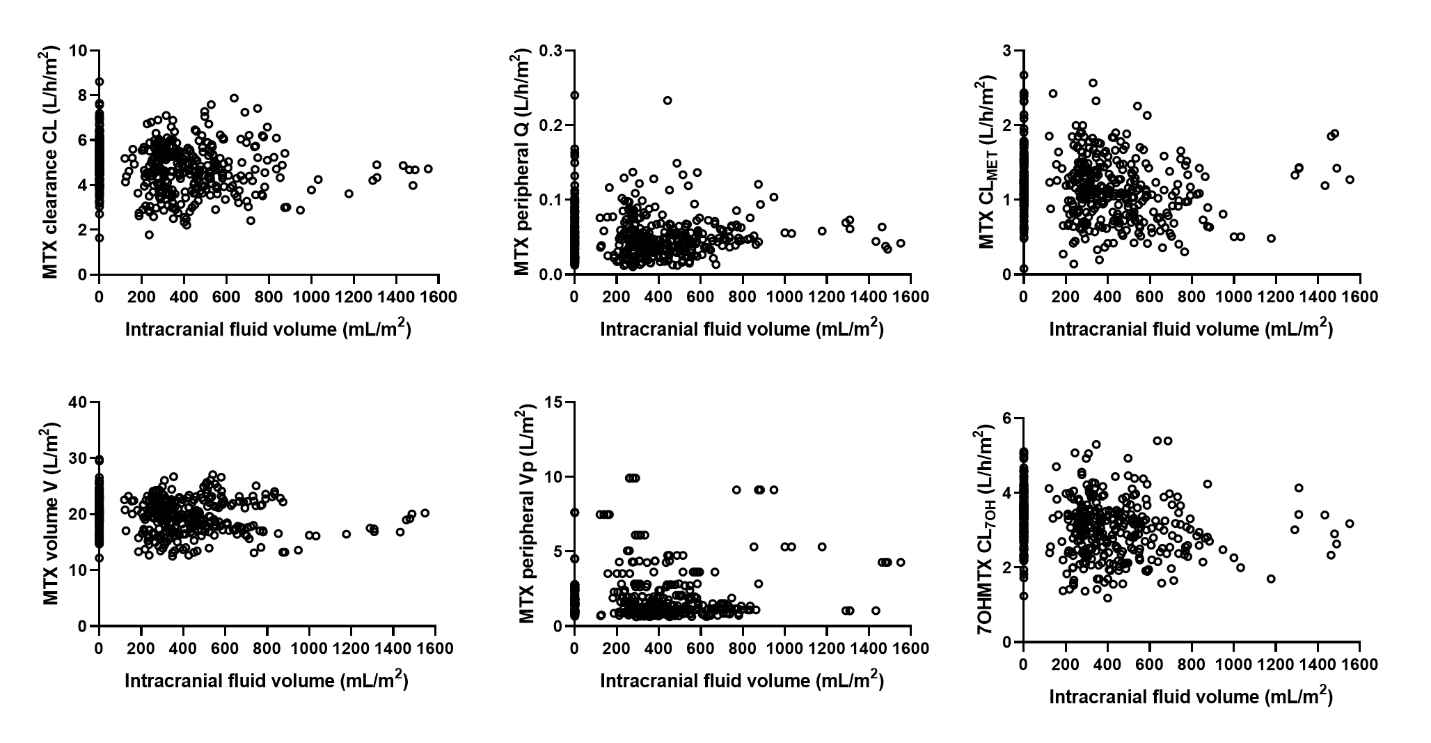


**Figure S7.** Prediction-corrected visual predictive checks for the final BSA-eGFR model.

In each graph, open-circles are the observed concentration-time data, and crosses are the concentrations below the limit of quantification. Solid lines represent the 50^th^ percentile of the model simulations. Shaded gray areas represent the 90% prediction interval (5^th^–95^th^ percentiles) of the model simulations.


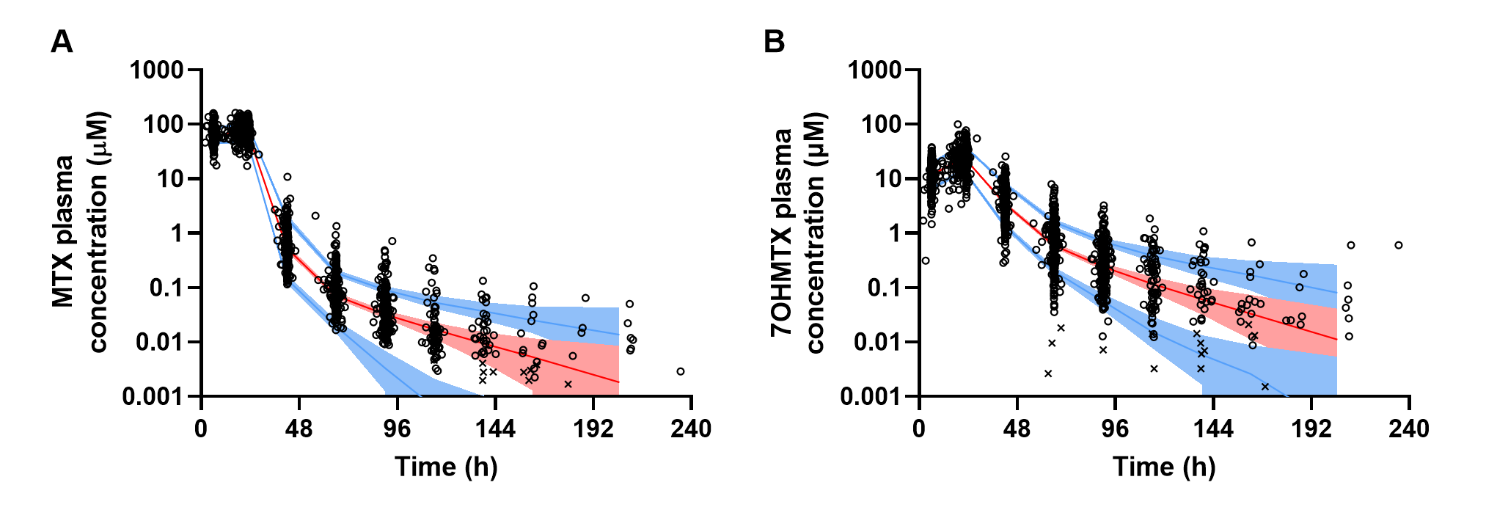


**Table S2.** MTX-related toxicities

| **Variable** | **Values** | **N** | **Percentage of all toxicities (N = 30)** | **Percentage of all MTX cycles (N = 513)** |
| --- | --- | --- | --- | --- |
| **Toxicity Grade** | 3 | 28 | 93.3 | 5.4 |
|  | 4 | 2 | 6.7 | 0.39 |
| **Attribution to MTX** | Possible | 25 | 83.3 | 4.87 |
|  | Probable | 4 | 13.3 | 0.78 |
|  | Definite | 1 | 3.3 | 0.19 |
| **Toxicity category** | Dermatology/Skin | 1 | 3.3 | 0.19 |
|  | Gastrointestinal | 27 | 90.0 | 5.3 |
|  | Infection | 1 | 3.3 | 0.19 |
|  | Metabolic/Laboratory | 1 | 3.3 | 0.19 |
| **Induction Cycle** | 1 | 11 | 36.7 | 2.1 |
|  | 2 | 8 | 26.7 | 1.6 |
|  | 3 | 3 | 10.0 | 0.58 |
|  | 4 | 8 | 26.7 | 1.6 |

**Table S3**. Characteristics of patients with methotrexate delayed excretion (DE) during cycles 2, 3, and 4.

| **Variable** | **Patients with DE at 42 hr** | **Patients with no DE at 42 hr** | **Patients with DE at 66 hr** | **Patients with no DE at 66 hr** |
| --- | --- | --- | --- | --- |
| **Cycle 2** |  |  |  |  |
| Age (years) | 1.60 ±0.94 | 1.99 ±1.0 | 1.90 ±1.11 | 1.86 ±0.89 |
| BSA (m^2^) | 0.50 ±0.12 | 0.54 ±0.10 | 0.53 ±0.12 | 0.53 ±0.10 |
| eGFR (mL/min/m^2^) | 125.4 ±37.1 | 117.3 ±26.4 | 122.3 ±33.1 | 117.6 ±27.5 |
| Intracranial fluid collections | | | | |
| N with / N without | 27 / 9 | 54 / 31 | 41 / 13 | 40 / 27 |
| Volumes (mL/m^2^) | 432.6 ±125.5 | 475.6 ±260.0 | 453.7 ±177.1 | 469.0 ±266.4 |
| **Cycle 3** |  |  |  |  |
| Age (years) | 1.48 ±1.1 | 2.15 ±0.92 | 1.89 ±1.2 | 2.05 ±0.81 |
| BSA (m^2^) | 0.48 ±0.11 | 0.56 ±0.09 | 0.51 ±0.11 | 0.56 ±0.09 |
| eGFR (mL/min/m^2^) | 112.5 ±32.8 | 116.7 ±24.4 | 114.7 ±27.9 | 116.3 ±25.9 |
| Intracranial fluid collections | | | | |
| N with / N without | 20 / 9 | 58 / 25 | 37 / 13 | 41 / 21 |
| Volumes (mL/m^2^) | 494.0 ±213.8 | 434.9 ±248.1 | 452.7 ±191.0 | 447.6 ±279.1 |
| **Cycle 4** |  |  |  |  |
| Age (years) | 1.76 ±1.2 | 2.19 ±0.89 | 2.10 ±1.12 | 2.07 ±0.85 |
| BSA (m^2^) | 0.50 ±0.13 | 0.56 ±0.08 | 0.53 ±0.12 | 0.55 ±0.09 |
| eGFR (mL/min/m^2^) | 107.9 ±20.8 | 110.6 ±21.9 | 109.4 ±21.5 | 110.5 ±21.8 |
| Intracranial fluid collections | | | | |
| N with / N without | 20 / 6 | 56 / 27 | 40 / 14 | 36 / 30 |
| Volumes (mL/m^2^) | 419.4 ±207.0 | 435.6 ±271.7 | 431.3 ±214.6 | 431.2 ±296.7 |

BSA, body surface area; eGFR estimated glomerular filtration rate

Data are reported as mean ± standard deviation

**Table S4.** Summary of pediatric MTX population PK studies from 1999 to 2023.

| **REFERENCE** | **DRUG(S)** | **Population** | **Dx** | **CL** | **CL & COVAR** | **COMMENTS** |
| --- | --- | --- | --- | --- | --- | --- |
| 1999, Odoul, Fundam Clin Pharmacol | HDMTX  8 g/m^2^ | 23 children  2 to 15 y | ALL | 3.5 L/h  (71.3 ± 45.3 ml/ min/m^2^) | Weight | Poor relationship betwn CL/Wt; Volume and age (CrCl was studied) |
| 2006, Aumente, Clin PK | HDMTX  3 g/m^2^ | 37 children  6 mo - 17 y | ALL  49 total | 5.3 L/h | TBW  ≤10 y 0.287  > 10 y 0.149 | TBW covar for CL and V; BSA 2^nd^ best covar; 12 in valid. Grp (SCr was studied) |
| 2007, Piard, CCP | HDMTX  5 g/m^2^ | 79 Children  2 to 16 y | ALL | 8.8 L/h | none | V and Wt (CrCl was studied) |
| 2008, Barrett, BMC Med Inform | HDMTX (dose N/S) | 240 patients  1 to 80 y | N/S | 8.1 L/h | N/A | N/A; example for error model for IIV; TDM (SCr was studied) |
| 2009, Colom, TDM | HDMTX avg 11.2 g/m^2^ | 14 children avg 14.8 y | OS | 4.8 L/h | Age and Wt | V and WT (CrCl was studied) |
| 2012, Rühs, Plos1 | HDMTX  1-4.6 g/m^2^ | 498 children med 5.4 y | ALL | 6.7 L/h | BSA and SCr (adj for age and gender) | Nothing for V (not purpose of study); homocysteine |
| 2013, EL Desoky, Pharmacol Pharm | HDMTX  2 g/m^2^ | 41 children  3 to 15 y | ALL | 2.18 L/h | BSA and sex | V and WT (CrCl was studied) |
| 2015, Jebabli, La Tunisie Medicale | HDMTX  1 to 8 g/m^2^ | 273 children  2 to 23 y | ALL | 8.72 L/h | CrCl, age, and Weight | Article in French, English abstract |
| 2015, Zhang, Chinese Med J | HDMTX 10 g/m^2^ | 148 AYA  17 ± 7 y | OS | 6.2 L/h | MTXNUM and CrCl | V and BSA; method to calculate CrCl N/S |
| 2019, Hui, J Clin Pharmacol | HDMTX  2-18 g/m^2^ | Children  1.3-19 y | 36 ALL &  16 OS | ALL –  7.73 L/h  OS –  5.2 L/h | ALL-BSA, CrCl; OS-Ht, CrCl, Dosage | SCr 0.1-1.4 mg/dl  ^CrCl 75-325 ml/min/1.73m; ALL-V -none; OS-V Ht |
| 2019, Beechinor, Clin Pharmacokin | HDMTX  4 g/m^2^ | 71 infants  < 366 days | ALL | 11 L/h/70 kg | Allometrically scaled WT (WT/70)^0.75^ | ****No SCr or CrCl included as covar; COG; Allometrically scaled WT (WT/70)^0.75^ |
| 2019, Zang, Int J Clin Pharmacol Therap [ABST] | HDMTX  Dose not specified | 190 children | ALL | 6.5 L/h | BSA | N/A; SCr |
| 2020, Shi, Biopharm Drug Disp | HDMTX  5 g/m^2^ | Children  0 to 15 y | BT (MB) | 9.23 L/h | CrCl, Wt, Dex (Y/N) | ^**CrCl 31-272 ml/min; apparent Vd fixed at 32.8 L |
| 2020, Medellin-Garibay, CCP | HDMTX  1-7 g | Children  1 to 15 y  (Mexican) | ALL-41 index; 9 validation | 6.0 L/h | BSA  No genotypes | V and TBW; CrCl 147±39 ml/min/1.73 – (Schwartz; 09); good genotype discussion; |
| 2020, Panetta, Br J Clin Pharmacol | HDMTX  5 g/m^2^ | 173 Infants & Young Children  0.02 to 4.7 y | MB | MTX – 92 ml/min/m^2^ | BSA, eGFR, *DEX & Vanc* | V and BSA, Dex, Vanc; eGFR 33 to 263 ml/min/m^2^ |
| 2020, Taylor, Clin Pharmacol Therap | HDMTX  0.6-10.1 g | 820 children 1-18.83 y | Ph- ALL | 11.0 L/h/ 1.73 m^2^ | Time-varying SCr (SCr_t_) |  |
| 2021, Gao, Front Pharmacol | HDMTX  3-5 g/m^2^ | 311 children  0.7-15y | ALL | 6.9 L/h | SCr | SCr .09-1.5 mg/dl; other PK parameters not related to covar. |
| 2023, Taylor, Clin Translat Sci | HDMTX  1-18 g/m^2^ | 1758 ped/AYA (0.1 to 32.3 y) | ALL, OS, and NHL | 8.24 L/h/ 1.73 1.73 m^2^ | BSA, SCr, infusion length, and Down Syndrome | Central volume, V2 – Pleural effusion  Intercompart CL, Q2, Q3 – hypoalb |
| 2023, Henz, Pharm Res | HDMTX 0.25-5 g/m^2^ | 45 ped pts (0.3 - 17.3 y) | ALL | 7.57 L/h | SCr, Ht, BUN, BMI (low) | Brazilian pediatric patients |

N/S: not specified; N/A: not available

Methods to calculate CrCl:

^Schwartz GJ, Pediatrics, 1976;

**Cockcroft, D. W., and Gault, M. H. (1976). Prediction of Creatinine Clearance from Serum Creatinine. Nephron 16 (1), 31–41. doi:10.1159/000180580;

^^Levey AS, Ann Intern Med, 2009 (CKD-EPI2009Scr)

@Traub formula
